# RIG-I-Like Receptors as Novel Targets for Pan-Antivirals and Vaccine Adjuvants Against Emerging and Re-Emerging Viral Infections

**DOI:** 10.3389/fimmu.2018.01379

**Published:** 2018-06-20

**Authors:** Hui Yee Yong, Dahai Luo

**Affiliations:** ^1^Lee Kong Chian School of Medicine, Nanyang Technological University, Singapore, Singapore; ^2^NTU Institute of Structural Biology, Nanyang Technological University, Singapore, Singapore; ^3^School of Biological Sciences, Nanyang Technological University, Singapore, Singapore

**Keywords:** RIG-I-like receptor, pan-antivirals, vaccine adjuvants, interferon, interferon-stimulated genes, RNA therapeutics

## Abstract

Emerging and re-emerging viruses pose a significant public health challenge around the world, among which RNA viruses are the cause of many major outbreaks of infectious diseases. As one of the early lines of defense in the human immune system, RIG-I-like receptors (RLRs) play an important role as sentinels to thwart the progression of virus infection. The activation of RLRs leads to an antiviral state in the host cells, which triggers the adaptive arm of immunity and ultimately the clearance of viral infections. Hence, RLRs are promising targets for the development of pan-antivirals and vaccine adjuvants. Here, we discuss the opportunities and challenges of developing RLR agonists into antiviral therapeutic agents and vaccine adjuvants against a broad range of viruses.

## Introduction

RNA viruses account for a third of all emerging and re-emerging infections (1). Due to the changes of abiotic and biotic landscape encountered by RNA viruses and the error-prone nature of viral replication, RNA viruses evolve quickly and contribute to the outbreak of infectious diseases (2). Many recent outbreaks of emerging and re-emerging viruses involve RNA viruses, and thus, there is an urgent need to develop antivirals against these viruses.

The innate immune system confronts viral infection *via* a specialized group of receptors known as pattern recognition receptors (PRRs) (3). Some PRRs recognize RNA viral infections including toll-like receptors 3 and 8 (TLRs), NOD-like receptors NLRP6 and 9 (NLRs), certain DDX/DHX helicases, and RLRs (retinoic acid inducible gene 1 (RIG-I) and melanoma differentiation associated protein 5 (MDA5)) (4–9). These PRRs usually activate interferon production and the secretion of pro-inflammatory cytokines (10). Interferon activates the Janus kinase/signal transducers and activators of transcription (JAK-STAT) signaling pathway in surrounding cells and the expression of interferon-stimulated genes (ISGs). ISGs inhibit virus replication and spread to surrounding cells by degrading viral nucleic acids and inhibiting viral gene expression (11, 12). Here, we focus on RLRs, the major sensors for pathogenic RNA species which trigger antiviral responses and discuss how modulation of RLRs may lead to broad-spectrum antivirals and new vaccine adjuvants.

## RIG-I-Like Receptors

RIG-I-like receptors are a class of DExD/H box RNA helicases which recognizes double-stranded RNA (dsRNA) (13–17). RLRs consist of RIG-I, MDA5, and laboratory of genetics and physiology 2 (LGP2) (18). RIG-I and MDA5 have similar structural domains with N-terminal caspase activation and recruitment domains (CARDs), central helicase domain, and C-terminal domain, which recognizes viral RNA ligands (19–21). RIG-I recognizes short dsRNA and binds to blunt-ended RNA with 5′ triphosphate moiety (22–27). In contrast, MDA5 binds to the stem region of longer dsRNA in a cooperative manner (28–30). LGP2, on the other hand, only have the helicase and C-terminal domain and are involved in the regulatory function of RIG-I and MDA5 (31, 32).

**Figure 1:**
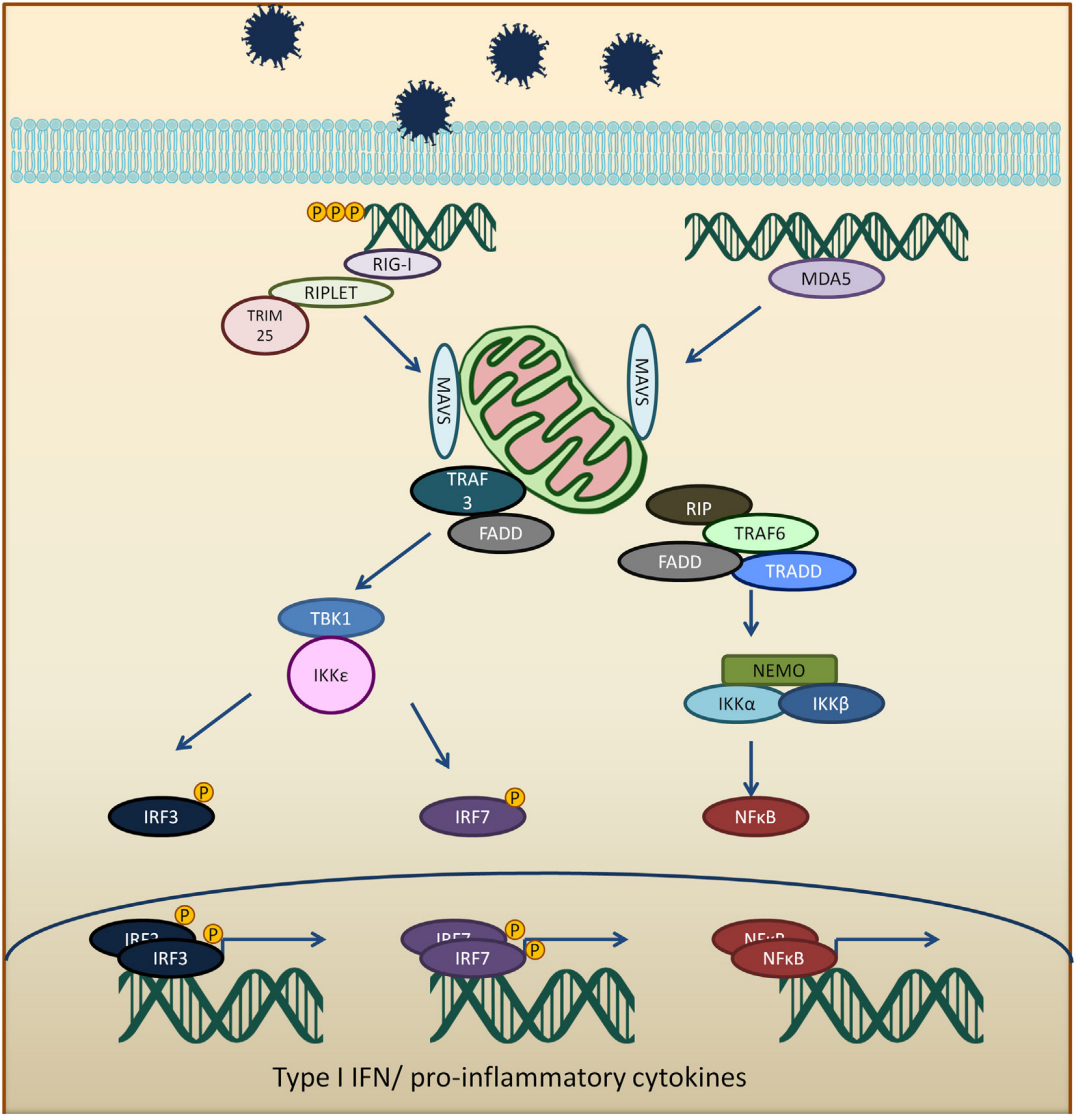
Viral RNA is recognized by RIG-I-like receptors (RLRs), RIG-I, or melanoma differentiation-associated protein 5 (MDA5). Activated RLRs interacts with mitochondria antiviral signaling protein (MAVS) adapter protein *via* CARD–CARD interactions. Activated MAVS then interacts with tumor necrosis factor receptor-associated factors 3 (TRAF3), tumor necrosis factor receptor-associated factors 6 (TRAF6), tumor necrosis factor receptor type-1-associated death domain (TRADD), receptor interacting serine/threonine-protein kinase 1 (RIP1), Fas-associated protein with death domain (FADD), and other signaling molecules. TRAF3 activates TANK binding kinase 1 (TBK1) and IκB kinase ε (IKKε), which phosphorylates interferon regulatory factors 3 and 7 (IRF3 and IRF7). The phosphorylated IRF3 and IRF7 dimerize and translocate into the nucleus to induce type 1 interferon response. On the other hand, MAVS interaction with receptor interacting serine/threonine-protein kinase 1, FADD, TRAF6, and TRADD. TRAF 6 ubiquitinate NF-kappa-B essential modulator (NEMO) which then activates IκB kinase and activates NF-κB. NF-κB transcription factor drives the expression of type 1 interferon and proinflammatory cytokines.

The CARD domains of RIG-I and MDA5 are involved in the activation of downstream signaling event *via* a protein known as mitochondria antiviral signaling protein (MAVS) (33–36). RIG-I binds to unanchored lysine-63 polyubiquitin chains and promotes efficient interaction with the CARD domain on MAVS (37, 38). MAVS protein polymerizes and forms fibrils when activated and will be polyubiquitinated and phosphorylated (38–42). The MAVS oligomer act as a platform to promote downstream antiviral signaling by recruiting several different proteins, such as tumor necrosis factor receptor type-1-associated death domain (TRADD), receptor interacting serine/threonine-protein kinase 1 (RIP1), Fas-associated protein with death domain (FADD), tumor necrosis factor receptor-associated factors (TRAF6, TRAF2, and TRAF3), as well as caspase 8 and caspase 10 (43, 44). TRAF3 activates TANK binding kinase 1/IκB kinase ε/IκB kinase γ/TANK (TBK1/IKKε/IKKγ/TANK) complex which phosphorylates and dimerizes interferon regulatory factors 3 and 7 (IRF3 and IRF7). The activated IRF3 and IRF7 translocate into the nucleus and activate IFN production (45, 46). TRAF 2 and 6 activate the IKKα/β/γ (also known as NEMO) by ubiquitination and resulting in activation of NFκB and the expression of pro-inflammatory cytokines (Figure 1) (41, 47).

## Pan-Antivirals Targeting RIG-I

Since RLRs are the key component for the antiviral immune response, these sensors are targets for antiviral therapeutics development. Current antiviral interventions focus on the use of direct-acting antivirals (DAAs), which target the essential components in the life cycle of a virus and thus are virus-specific (48). Although DAAs are highly effective, the low fidelity replication of the RNA virus genome could ultimately lead to the emergence of DAA therapies escape mutant (49). To circumvent this problem, broadly targeting antiviral therapeutics need to be used synergistically with DAAs. To this end, RIG-I agonists or RIG-I pathway activators represent a novel group of promising antiviral candidates. Lists of the antiviral candidates are discussed below as three categories based on their chemical nature (Table 1).

**Table 1:** Pan-antivirals targeting RLRs.

Pan-antivirals	Formula	Target pathway	Reference
**Nucleotide-based**			

SB9200	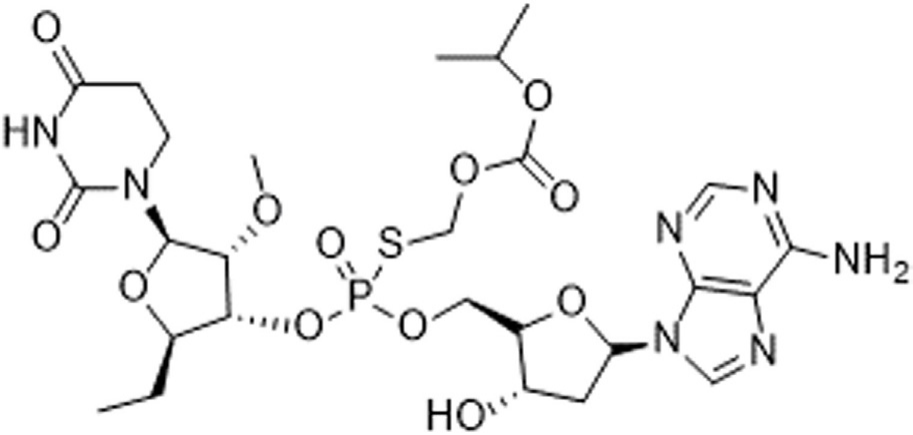	RLR and NLR	(50–52)

**RNA-based**			

5′ppp RNA derived from the 5′ and 3′ UTRs of the negative-strand RNA virus Vesicular Stomatitis Virus	GACGAAGACC ACAAAACCAG AUAAAAAAUA AAAUUUAAUG AUAAUAAUGG UUUGUUUGUC UUCGUC	RLR	(53, 54)

5′ppp RNA (M8)	GACGAAGACCACAAAACCAGAUAAAAAAAAAAAAAAAAAAAAAAAAAAUAAU UUUUUUUUUUUUUUUUUUUUUUUUUUAUCUGGUUUUGUGGUCUUCGUC	RLR	(55)

5′OH RNA with kink (CBS-13-BPS)	GGUAGACGAAACCAGAUAUAAUAUCUGGUUUCGUUUGCC	RIG-I, ISG56	(56)

5′PPP SLR	Stem loop RNA with the length of 10 and 14 base pair (GGACGUACGUUUC GACGUACGUCC) and (GGAUCGAUCGAUCGUUCGCGAUCGAUCGAUCC)	RIG-I	(57)

**Small molecular compounds**			

KIN 100	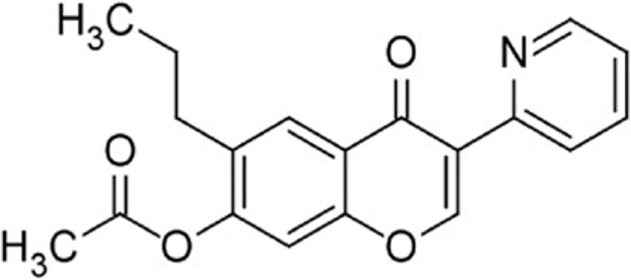	IRF 3	(58)

KIN101	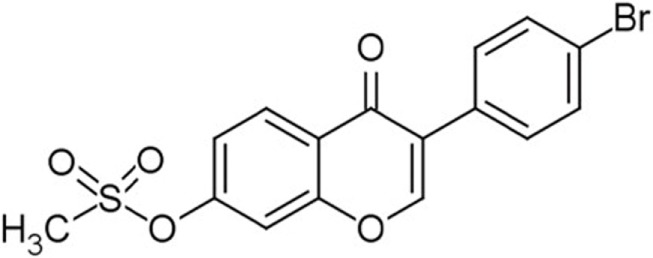	IRF3	(58)

KIN 1000	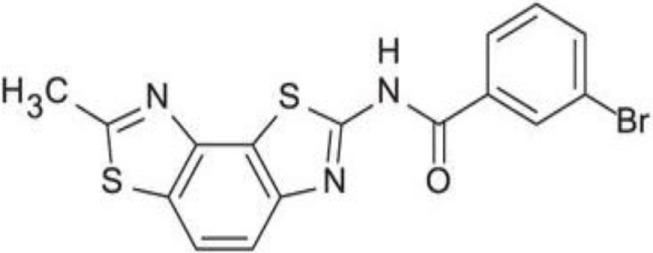	IRF 3	(59)

KIN1400	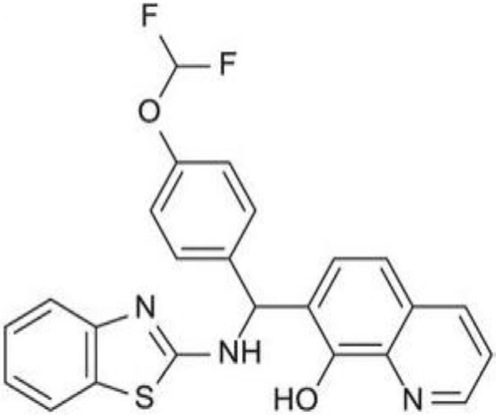	IRF 3	(59)

KIN1408	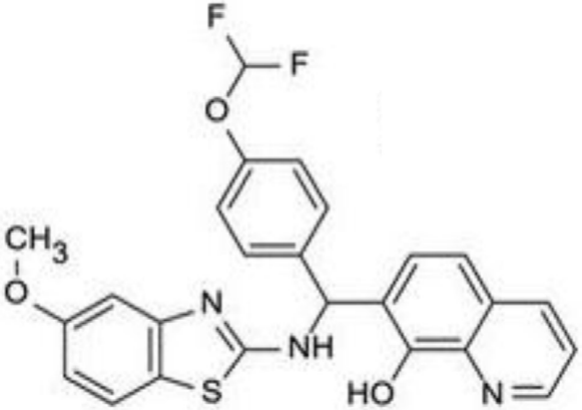	IRF3	(59)

KIN1409	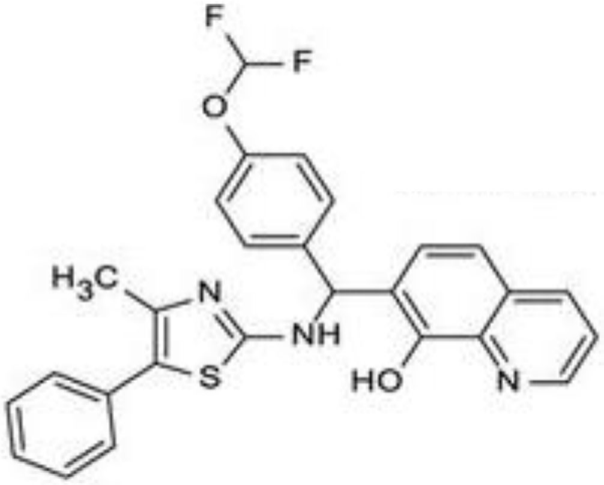	IRF3	(59)

*RLRs, RIG-I-like receptors*.

## Nucleotide-Based Antivirals

A dinucleotide-derived small molecule compound, SB9200, has been shown to induce IFN *via* RIG-I and nucleotide-binding oligomerization domain containing protein 2 (NOD2). SB9200 is believed to interact with RIG-I and NOD2 that are associated with pre-genomic RNA thus blocking the HBV viral polymerase from replicating the genomic RNA (60). SB9200 was shown to confer dose-dependent and long-lasting induction of IFNα, IFNβ, and ISGs in liver tissue (50). Treatment of this compound in woodchucks infected with Woodchuck Hepatitis Virus (WHV) showed no sign of toxicity with reduced hepatic WHV antigen and nucleic acid. The sequential treatment of WHV-infected woodchuck with SB9200 followed by entecavir (ETV), a currently used antiviral to treat Hepatitis B (HBV), showed a reduction in viremia and delayed recrudescence of viral replication. The viral reduction from the treatment of SB9200 was comparable with current antivirals, such as Emtricitabine, Tenofovir, and Adefovir, when administered for up to 12 weeks (51). Drugs such as Emtricitabine, Tenofovir, and Adefovir are commonly used for the treatment of HBV. These drugs, however, may cause side effects such as lactic acidosis and possible liver, and kidney failure. SB9200 is effective for HCV patients with relapse after DAA and interferon treatment and could serve as a promising treatment option for patients who are not responding to the current regimen of DAA therapy (52). The phase 1 clinical trial on naïve adult with chronic hepatitis C showed an association between the decline in viral RNA and the peak of SB9200 detection in plasma (Clinical trial no NCT01803308). Currently, SB9200 is being tested in phase 2 clinical trials for treating subjects chronically infected with the HBV.

## RNA-Based Antiviral Candidates

5′ triphosphorylated and diphosphorylated short dsRNAs are RIG-I specific ligands (22, 26, 61, 62). Goulet et al. showed that 5′pppRNA could activate a broad spectrum of antiviral and inflammatory genes such as IRF3, IRF7, NFkB, and the downstream ISGs. Treatment of lung epithelial cells A549 with 5′pppRNA confers protection against vesicular stomatitis virus (VSV), vaccinia virus, and dengue virus (DENV). The antiviral effect of 5′pppRNA was also detected against HIV in CD4+ T cells and HCV in Huh 7.5 cells (53). Besides that, 5′pppRNA was also an effective antiviral against influenza virus infection *in vitro* and *in vivo*. Treating mice with 5′pppRNA prior to influenza virus challenge also reduces pneumonia related to influenza virus infection (53). In another study, 5′pppRNA was shown to stimulate host antiviral response and reduce the infectivity of DENV and chikungunya virus (CHIKV) in human myeloid, fibroblast, and epithelial cells *via* RIG-I specific activation (54).

Several studies were also carried out to determine factors such as sequence, length, and structure of 5′pppRNA to enhance the antiviral activities of RIG-I (55, 56). Chiang et al. showed that 5′pppRNA with uridine-rich sequence with 99 nucleotides hairpin (M8) triggered higher interferon response when compared to other RIG-I aptamer and poly(I:C). M8 specifically activates RIG-I without triggering MDA5 or TLR3 activation. Prophylactic and therapeutic treatment using M8 protect cells from dengue and influenza viral infections. Furthermore, administration of M8 followed by influenza virus challenge improves the survival rate of mice with low lung virus titer detected at day 3 post-infection (55). In another study carried out by Lee et al., different RNA fold was shown to elicit different antiviral properties *via* RIG-I. Short hairpin RNA with a bent in the stem structure with phosphorothioate backbone was used as antiviral and was more potent than oseltamivir against influenza A H1N1 virus *in vitro* (56). Linehan et al. recently showed that short RNA with stable tetraloop at one end of duplex RNA triggers a robust IFN 1 response *in vivo*. These short stem-loop RNA (SLR) induces a subset of genes involved in antiviral and effector responses as well as represses gene involved in T cell maturation and could potentially be developed into a highly effective antiviral or vaccine adjuvant (57).

## Small Molecular Compounds

High throughput screening (HTS) of small molecule compounds identified a group of novel agonists of the innate immune pathway. The isoflavone-like compound confers protection against HCV and Influenza A virus *in vitro*. These compounds were also shown to activate a narrower subset of genes and thus have potential to be useful antiviral without causing cytokine toxicity (58).

Another class of small molecule compounds, hydroxyquinolines, identified *via* HTS of compound library in cell culture induces the expression of innate immune antiviral genes such as RIG-I, IFIT1, IFIT2, IFITM1, OAS3, and MX1. Remarkably, although these compounds were able to induce high expression of antiviral genes, the expression of type I and III interferon remains low, suggesting the activation of distinct antiviral pathway than that of RIG-I agonists. The specific target(s) of these hydroxyquinoline compounds are not known. These hydroxyquinoline compounds were effective antivirals against a broad range of RNA viruses from the families Flaviviridae, Filoviridae, Paramyxoviridae, Arenaviridae, and Orthomyxoviridae. Interestingly, these compounds exhibit both prophylactic and therapeutic activity against infection and could be used in combination with other antivirals (59).

## Innate Immune Potentiator as Vaccine Adjuvants

**Figure 2:**
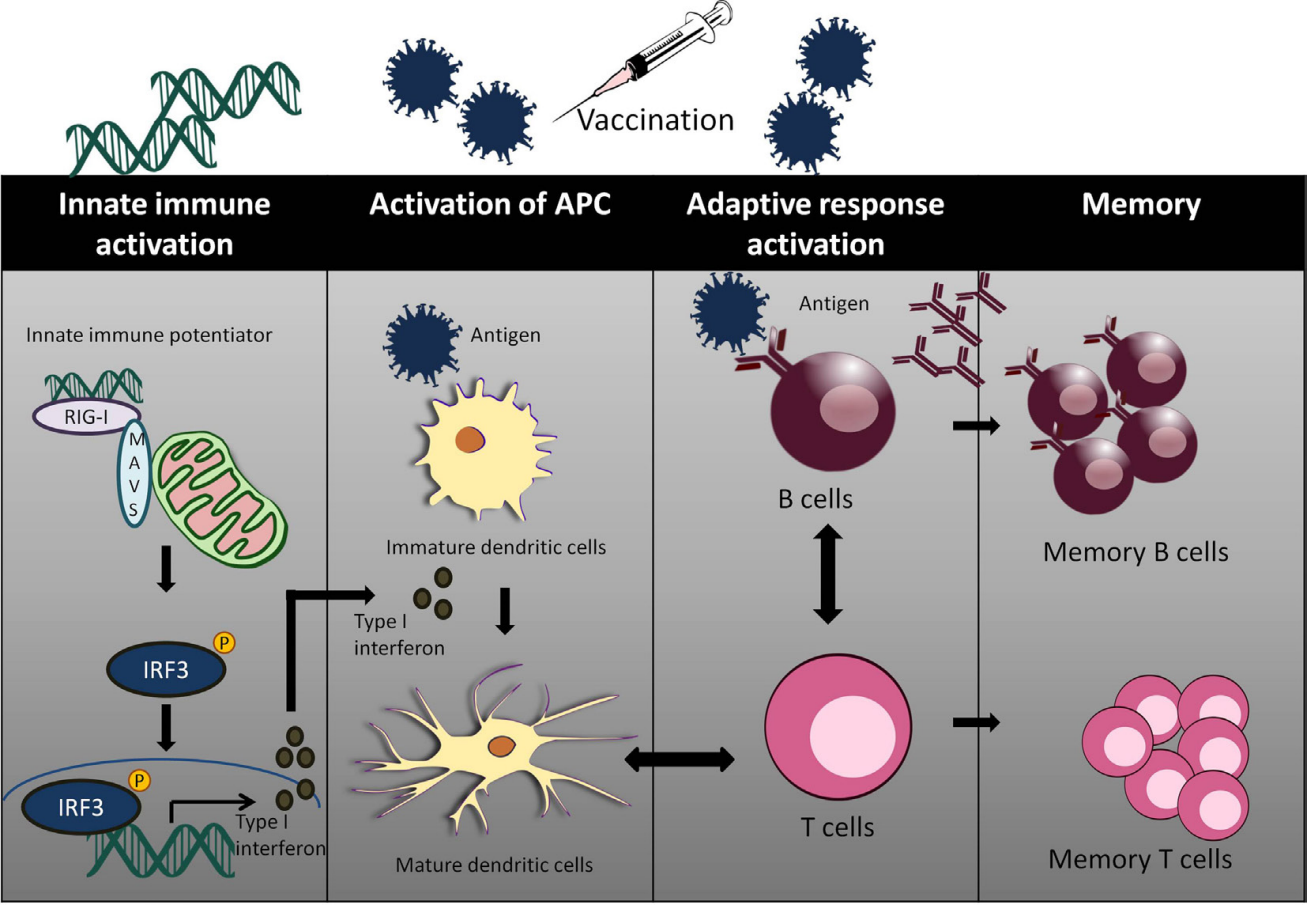
The use of innate immune potentiator as adjuvant triggers the stimulation of adaptive immune responses. Innate immune potentiator stimulates RIG-I-like receptors (RIG-I) and interacts with MAVS adapter. This results in the activation of downstream signaling pathways and release of type I interferon. Type I interferon couple with the presence of antigen trigger DC maturation by enhancing surface marker expression and antigen presentation. The activated DCs interact with CD4+ T cells and thus stimulate Type 1T helper (TH1) cells. TH1 cells in turn interact with B cells to produce antibodies and trigger clonal expansion of B cells and T helper cells.

Adjuvants act as an immune enhancer to a vaccine. Several different classes of adjuvant had been approved for use in human vaccines such as alum and oil in emulsion MF59, AS03 (oil in water emulsion), virosomes, and AS04 (aluminum with monophosphoryl lipid A) (63). Alum and MF59 act by increasing antigen uptake at the injection site and activates pro-inflammatory responses (64–66). Alum mainly acts *via* Th2 cellular immune response, which does not confer the best protection for viral infections such as HCV and HIV. Moreover, there are safety concerns with the use of alum as an adjuvant with reported cases of hypersensitivity and erythema (67, 68). Well characterized agonists of innate immunity may serve as a better candidate of targeted vaccine adjuvants (Figure 2).

A small molecule compound named KIN 1148, discovered *via* HTS, was shown to activate IRF3 nuclear translocation. When this compound was tested with influenza split virus vaccine H1N1 A/California/07/2009, it confers protection from lethal challenge of influenza virus strain A/California/04/2009. KIN1148 together with the vaccine confers protection *via* IL-10 and Th-2 response to T cells in lung and lung-draining lymph nodes. Immunization with vaccine and KIN 1148 showed a significant increase in IgG antibodies with serum from mice receiving prime-boost immunization conferring protection to naïve mice from influenza challenge. KIN1148 was shown to be able to work alongside the vaccine to boost protective immunity and protect against influenza strain A/California/04/2009 (Table 2) (69).

**Table 2:** Innate immune potentiator as virus vaccine adjuvants.

Adjuvant	Target	Status	Virus vaccine	Reference
KIN 1148 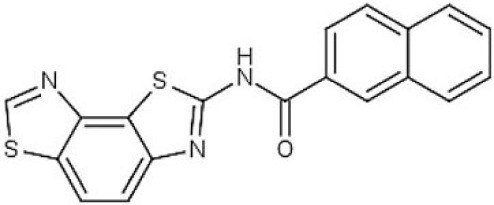	IRF3	Laboratory testing	Influenza H1N1 A/California/07/2009	(69)

M8 5′pppRNA GACGAAGACCACAAAACCAGAUAAA AAAAAAAAAAAAAAAAAAAAAAAUA AUUUUUUUUUUUUUUUUUUUUUU UUUUUAUCUGGUUUUGUGGUCUUCGUC	RLR	Laboratory testing	H5N1 influenza	(70)

5′pppRNA Derived from SeV DI RNA	RLR	Laboratory testing	H1N1 2009	(71)

*RLRs, RIG-I-like receptors*.

5′triphosphorylated duplex RNA was tested as an adjuvant by Beljanski et al. M8 a potent triphosphorylated RNA was used in conjunction with virus-like particle (VLP) expressing H5N1 influenza hemagglutinin and neuraminidase. The combination of VLP and RNA increases the survival rate of mice infected with H5N1 influenza virus and induces higher antibody titer against influenza virus as compared to other adjuvants such as alum, addavax and poly(I:IC). Furthermore, vaccination with VLP and RNA stimulates TH1-biased CD4 T cells response in mouse sera (70). Another 5′triphosphorylated RNA derived from Sendai virus defective interfering RNA (SeV DI RNA) was also tested as adjuvant together with the H1N1 2009 pandemic vaccine and was shown to enhance production of influenza-specific IgG antibodies and influenza-specific IgA antibodies indicating that this 5′triphosphorylated RNA could potentially be used as influenza vaccine adjuvant (71).

## Delivery of RNA-Based Agents Remains Challenging

To be effective as therapeutics, functional RNA species must be internalized into targeted cells. The delivery methods commonly used for RNA-based or nucleotide specific antivirals includes the lipid-oligo complexes, nanoparticle-based delivery, and viral-based delivery. The hydrophilic, negatively charge nature of RNA hinders the direct uptake of naked oligos into cells. The administration of RNA *via* inhalation was poorly efficacious in lung macrophages and only intratracheal administration leads to efficient delivery of RNA into the targeted site (72). Besides suffering from poor uptake, naked RNA is often prone to degradation in plasma (73).

One favored method of delivery for RNA is lipid-oligo complexes. This delivery method is more efficient due to the tendency of lipid to interact with the cell membrane and improve the uptake of RNA (74). A biocompatible lipid-based carrier can further reduce undesired immunogenic activation. However, the cationic nature of lipid is reported to interact with proteins in serum, and these aggregates are cleared by organs such as the spleen, lung and liver (75).

Nanoparticle-based delivery is a versatile method for RNA delivery with many organic or inorganic materials available as nanocarriers. Nanoparticle requires less RNA material with a large surface area for interaction with cells (76). The material used as a carrier could also be tailored for application such as dose, circulation time, as well as passive or active release of RNA (77). To overcome the issue of toxicity or uncontrolled immune activation, multistage delivery of RNA from nanoparticles could be carried put (78). The downside of this strategy is the need to test various materials for RNA delivery and this would drive up the cost of therapeutics or vaccine.

## The Danger of Uncontrolled Immune Activation Always Exists

The therapeutic and prophylactic use of pan-antivirals was previously demonstrated in viral infection of influenza and dengue (55, 69, 71). Hotz et al. demonstrated that the pre-exposure of murine APC to synthetic poly(I:C) inhibits RLR activation while augmenting the sensitivity of TLRs. This would also imply a narrow therapeutic window for the use of pan-antiviral RNA targeting RIG-I (79). For clinical usage, the dosage of therapeutics is important to minimize side effects such as exacerbated cytokine storms and toxicity. Prater et al. showed that the injection of pregnant C57BL/6 mice with a high dose of CpG-ODN resulted in high fetal resorptions and craniofacial/limb defects (80). RIG-I agonists face similar concerns.

## RIG-I SNPs may Lead to Poor or Hyper-Responsiveness

There are 324 RIG-I SNPs identified from NCBI SNPs database with 8 resulting in amino acid changes or truncation. The S183I mutation on RIG-I weakened the antiviral signaling and produces a low level of IFNβ and NFκB upon IAV and SeV challenges in a cell-based assay. Another SNP of RIG-I at P229 resulted in frameshift mutation at the CARDs domain and triggers constitutive expression of IFNβ suggesting that individual with this mutation could be linked to hyper-responsiveness in the immune system or autoimmune diseases (81). Another commonly found SNP (rs10813831) of RIG-I resulted in the substitution of R7C which could alter RIG-I interaction with MAVS (82). Individuals with these SNPs have a lower rubella-specific IgG titer when immunized with live measles-mumps-rubella (MMR-II) vaccine (83). This SNPs mutation also increased the IFNβ level and RIG-I transcription in human dendritic cells when infected with Newcastle disease virus (NDV) (82). Individuals with these SNPs were also shown to have complications of brainstem encephalitis when infected with enterovirus 71 (84). Several intronic SNPs also alter the cellular and humoral response to the measles vaccine. Genotype of individual carrying the SNP in minor allele of RIG-I (for rs12555727, rs12006123, and rs17289116) also showed less virus-specific IFN-γ secretion against measles. These findings imply that genetic variants are also involved in initial antiviral responses to vaccination (85) The haplotype of RIG-I rs3739674 which is located in the 5′UTR is associated with higher EV71 HFMD risk possibly by altering the expression level of the gene (86). In order to target RIG-I as pan-antiviral or vaccine adjuvant, the different haplotypes affecting the disease outcome should be considered. Dosage concern should be taken into account to enhance the effectiveness of RIG-I as a broadly targeting antiviral or vaccine adjuvant.

## Conclusion

Emerging and re-emerging viruses present a significant public health concern, and there is an urgent need for novel vaccination and treatment strategies. RIG-I agonists as new adjuvant candidates may work alone or couple to vaccine agents such as VLPs or recombinant proteins to improve the safety and efficacy of conventional vaccines. Antivirals targeting the innate arm of immunity (host-directed therapy) would be useful to confer protection against emerging and re-emerging viruses (87). However, the development of such vaccines and antivirals is still in its infancy and many challenges related to the production and safety evaluation of vaccines and antivirals. Several key issues still need to be addressed including production platform, formulation, delivery, safety, and the ability of such class of the vaccine adjuvant or antivirals to be used in immunocompromised and elderly.

## Author Contributions

DL and HY discussed, wrote, and revised the manuscript.

## Conflict of Interest Statement

The authors declare that the research was conducted in the absence of any commercial or financial relationships that could be construed as a potential conflict of interest.
